# Improvement of computational fluid dynamics simulations of flow in patients with total cavo pulmonary connection and predicting interventional outcomes

**DOI:** 10.1186/1532-429X-18-S1-O72

**Published:** 2016-01-27

**Authors:** Petter Frieberg, Petru Liuba, Pia Sjöberg, Einar Heiberg, Johan Revstedt, Håkan Arheden, Marcus Carlsson

**Affiliations:** 1 grid.4514.40000000109302361Department of Clinical Physiology, Skåne University Hospital, Lund University, Lund, Sweden; 2 grid.4514.40000000109302361Dept. of Pediatric Cardiology, Lund University Hospital, Lund University, Lund, Sweden; 3 grid.4514.40000000109302361Department of Energy Sciences, Faculty of Engineering, Lund University, Lund, Sweden

## Background

Computational Fluid Dynamics (CFD) can be utilized to evaluate hemodynamic characteristics in patients with surgically implemented Total Cavo Pulmonary Connection (TCPC). The simulation process is however often complex and rarely takes into account characteristics such as pulmonary resistance and Aorto-Pulmonary Collaterals in analyses to predict outcome of interventions. The aim of this study was therefore to develop a framework to predict interventional outcomes on TCPC patients by using commercial off-the-shelf software for patient-specific CFD simulations including pulmonary resistance and the effect of Aorto-Pulmonary Collaterals.

## Methods

Patient-specific reconstructions of TCPC vessels (n = 11) were constructed by importing CMR segmentations into a 3D-design software, where a continuous 3D model was formed on the anatomical boundaries. Fluoroscopy angiography images were superimposed on the 3D model to aid reconstruction in areas where segmentation were unavailable due to stenting-induced CMR artifacts (n = 3). In the CFD software, pulmonary resistance was simulated using porous properties in the distal pulmonary arteries. Time-averaged 2D phase contrast (2D-PC) CMR flows were used as inlet boundary conditions. Static pressure was used as outlet boundary conditions, simulating atrial pressure. When CMR showed greater pulmonary venous return than was provided by the corresponding pulmonary artery, indicating Aorto-Pulmonary Collaterals, the throttling effect of the differential flow was included in the simulation. CFD results were compared to 2D-PC results, and when available, 4D-PC results. Interventions in two patients with a stent-dilation and a y-graft surgery respectively, were modeled and compared to post-interventional CMR results.

## Results

**Figure 1:**
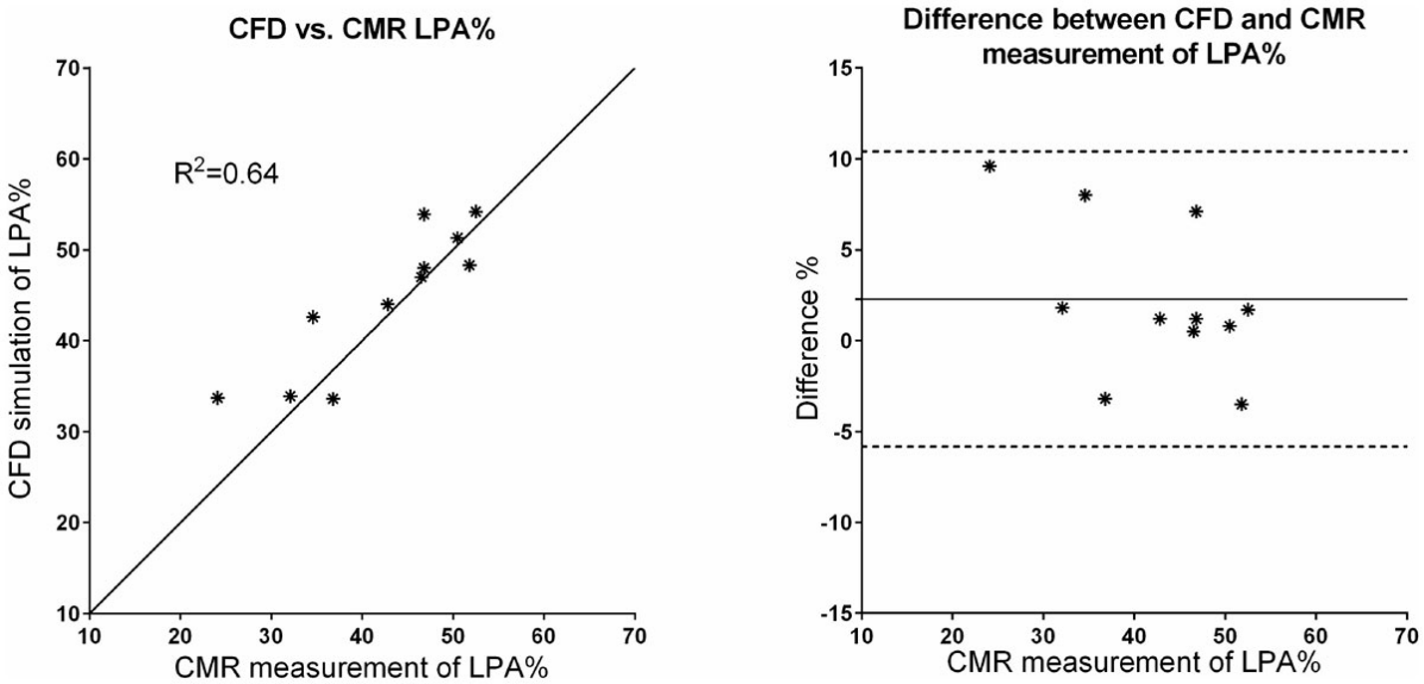
**Correlation and Bland-Altman plot for CFD simulation against CMR measurement of flow ratio to Left Pulmonary Artery (LPA%)**. CFD simulation of LPA% plotted against identity line of CMR measurement of LPA% (left) and difference between CFD simulation and MRI measurement of LPA% (right).

**Figure 2:**
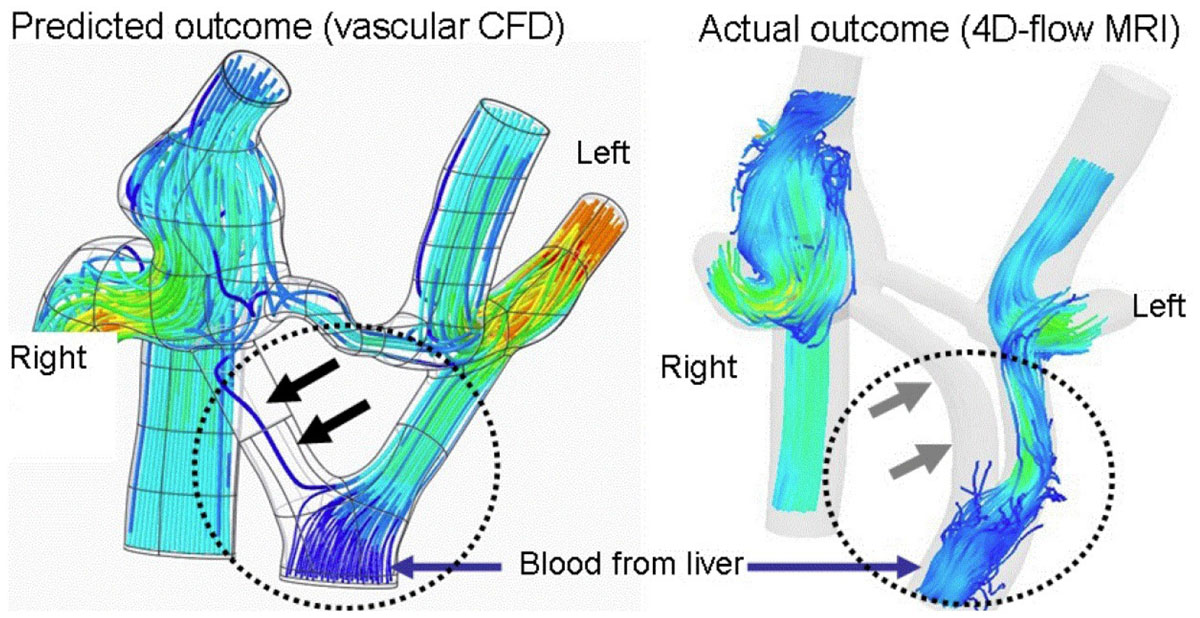
**A Fontan patient where modeling, based on patient specific image data, could have predicted the surgical outcome**. Surgery would not have been performed in this patient if the predicted results had been known before the intervention. The left image shows the modeled flows within the vessels delivering blood to the lungs. A y-shaped graft (dashed circle) was surgically implanted to divide the flow from the liver to both lungs. The model predicted that flow to the right lung in the inserted y-graft would be near zero (black arrows) and that surgery therefore was unsuccessful. 4D-flow on a post-operative CMR (right image, grey arrows) confirmed the vascular CFD modeling results.

The difference between simulated and actual flow ratio between Left and Right Pulmonary Artery (LPA% and RPA%, respectively) according to Bland-Altman analysis (Figure 1) was 2.3 ± 4.1%. In patients where significant Aorto-Pulmonary Collaterals were found (n = 6), including these in the simulation reduced simulation error from 9.0 ± 7.3% to 4.2 ± 4.5%. Analysis of the patient with stent-dilatation showed 32.3% before and 33.9% LPA flow after stent dilatation. CMR 2D flow showed similar results (30.2% pre- and 32.1% post-dilatation). CFD modelling of the surgical y-graft replacement (Figure 2) correctly predicted that it had little effect on its desired outcome to split the hepatic inflow to both lungs. Post-surgical percentage hepatic flow to LPA was 100% on 2D and 4D-flow CMR and simulated percentage was 100%.

## Conclusions

This study has demonstrated that the introduction of porous properties to simulate pulmonary vascular resistance and including Aorto-Pulmonary Collateral flow in the CFD analysis improves the accuracy to predict flow in TCPC vessels. Preliminary findings in two patients show that the effect of surgical and catheter interventions could be predicted using CFD analysis.

